# Anesthetic management of patients undergoing cardiac myxoma resection: a single-center retrospective analysis

**DOI:** 10.3389/fcvm.2023.1126822

**Published:** 2023-04-27

**Authors:** Wei Qi, Xiao-lu Yu, Da-xuan Yang, Xu-kai Hu, Jun-ping Chen, Yun-tai Yao, the Evidence in Cardiovascular Anesthesia (EICA) Group

**Affiliations:** ^1^ Department of Anesthesiology, Ningbo No.2 Hospital, Zhejiang, China; ^2^ Department of Anesthesiology, Fuwai Hospital, National Center for Cardiovascular Diseases, Peking Union Medical College and Chinese Academy of Medical Sciences, Beijing, China; ^3^ Department of Gynecology, Ningbo Women and Children’s Hospital, Zhejiang, China

**Keywords:** cardiac myxoma, myxoma resection, anesthetic management, mitral valve obstruction, hemodynamic instability

## Abstract

**Background:**

Myxomas are the most common primary cardiac tumors. Intracardiac myxomas, although benign, could cause serious consequences such as tricuspid or mitral valve obstruction, hemodynamic collapse, and acute heart failure, which pose challenges during anesthetic management. The current study was designed to summarize the anesthetic management of patients undergoing cardiac myxoma resection.

**Methods:**

This study was performed retrospectively from the perioperative period of patients who underwent myxoma resection. Patients were divided into two groups according to whether the myxoma prolapsed into the ventricle (group O) or not (group N) to evaluate the impact of tricuspid or mitral valve with obstruction.

**Results:**

110 patients, aged 17–78 years, undergoing cardiac myxoma resection between January 2019 and December 2021 were collected, and their perioperative characteristics were recorded. In the preoperative evaluation, common clinical symptoms included dyspnea and palpitation, whereas embolic events occurred in 8 patients, including 5 (4.5%) cerebral thromboembolic events, 2 (1.8%) femoral artery, and 1 (0.9%) obstructive coronary artery. According to the echocardiography, left atrial myxoma was detected in 104 (94.5%) patients, the average dimension of myxoma was 4.03 cm ± 1.52 cm in the largest diameter, and 48 patients were divided into group O. During intraoperative anesthetic management, hemodynamic instability occurred in 38 (34.5%) patients after anesthesia induction. More patients in group O had hemodynamic instability (47.9% vs. 24.2%, *p* = 0.009) than in group N. The mean postoperative length of stay in the hospital was 10.64 ± 3.01 days, and most of the patients made an uneventful postoperative recovery.

**Conclusions:**

Anesthetic management for myxoma resection can be composed by assessing the myxoma, particularly the echocardiography evaluation and preventing cardiovascular instability. Typically, tricuspid or mitral valve with obstruction is a premier ingredient in anesthetic management.

## 1. Introduction

Cardiac myxomas are the commonest type of cardiac tumor, accounting for nearly 50% of primary intracardiac lesions (1, 2). Although cardiac myxomas are histologically benign, they may be lethal because of their variable position (1, 3). Meanwhile, even sporadic myxomas can pose many anesthetic challenges, such as tricuspid or mitral valve obstruction (4), hemodynamic instability (5), acute heart failure, and death (6), which makes anesthetic management quite a challenge.

However, the current literature contains only sparse recommendations for anesthetic management (5, 7). Therefore, the anesthetic management needs to be clarified for patients undergoing myxoma resection, especially the prevention of hemodynamic instability after induction of general anesthesia.

In this article, our anesthetic management experiences in 110 cases of primary cardiac myxoma resection from the Chinese population are presented to emphasize the characteristics of myxomas, especially the prevention of cardiovascular instability.

## 2. Methods

### 2.1. Ethical approval

The study was approved by the Ethical Committee of Fuwai Hospital (2019-1301). Because of the retrospective nature of this study, patient consent was waived.

### 2.2. Study design and group

**Figure 1:**
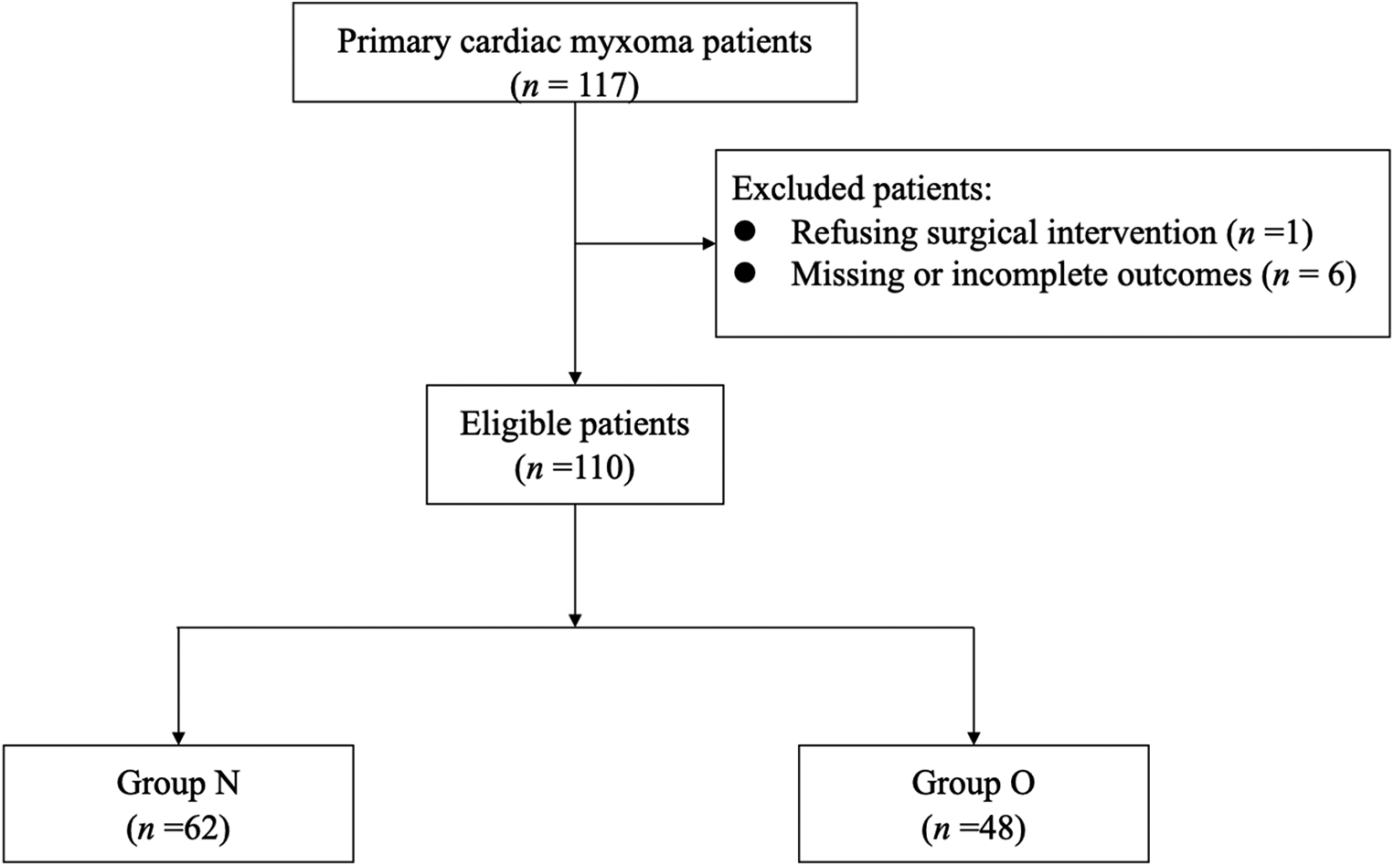
Patient inclusion.

This study was performed retrospectively from the perioperative period of patients who underwent myxoma resection, including the preoperative evaluation, intraoperative anesthetic management, and postoperative outcomes. Between January 2019 and December 2021, 117 patients were diagnosed with primary intracardiac myxoma at Fuwai Hospital. Exclusion criteria included: (1) patients refusing cardiac surgery; (2) patients whose outcomes of interest were missing or incomplete. Included patients were divided into two groups according to whether the myxoma prolapsed through the tricuspid valve (TV)/mitral valve (MV) into the right ventricular (RV)/left ventricular (LV) (group O) or not (group N) (Figure 1). These operations constituted 0.28% of the 39,045 cardiac surgical procedures performed in this center during that period.

### 2.3. Preoperative evaluation

For the preoperative evaluation, routine physical examination and hematology, biochemistry, chest x-ray (CXR), and electrocardiographic studies were performed in all patients. The diagnosis was established in all patients by two-dimensional transthoracic echocardiography (TTE). Meanwhile, according to TTE results, myxomas were divided into group O or group N to evaluate myxoma's impact, which prolapsed into the ventricle and obstructed the tricuspid or mitral valve.

### 2.4. Anesthetic management

The primary intracardiac myxoma resection was performed under general anesthesia in all patients through median sternotomy accompanied by moderate hypothermia and cardiopulmonary bypass (CPB). Standardized intraoperative monitoring for cardiovascular surgical includes electrocardiogram (ECG), arterial blood pressure (ABP), central venous pressure (CVP), pulse oximetry (SpO_2_), capnography (ETCO_2_), body temperature and transesophageal echocardiography (TEE). Arterial catheterization for ABP monitoring was performed before anesthesia induction. The general anesthetic technique was standardized, which included sufentanil 2–3 µg/kg, etomidate 2 mg/kg, cis-atracurium 0.15–0.2 mg/kg for anesthesia induction, and sevoflurane 0.5%–2.5%, dexmedetomidine 30–50 µg/h, propofol 100–200 mg/h, and cis-atracurium 10–20 mg/h for anesthesia maintenance. Sufentanil 1 µg/kg was added before CPB, during CPB inhalation anesthesia was stopped and intravenous anesthesia was maintained, and Sufentanil 1 µg/kg was added again after rewarming. Adequate anesthesia depth was achieved by maintaining the bispectral index (BIS) values between 40 and 60 intraoperatively (8).

Hemodynamic instability was defined as a fall in the systolic blood pressure of ≥30% from baseline or ≤80 mmHg for >1 min after induction. An intermittent bolus of methoxamine, phenylephrine, and atenolol, and intravenous infusion of dopamine and or milrinone were administered to maintain intraoperative hemodynamic stability, which responsible anesthesiologists determined.

Heparin was injected 400 U per kilogram after skin incision to achieve heparinization. Failure to meet the target activated clotting time (ACT) of more than 410 s, an extra 100–200 U per kilogram heparin was repeated and diagnosed as heparin resistance.

### 2.5. Postoperative outcomes

All patients were admitted to the intensive care unit (ICU) after surgery. The extubation decision was achieved if patients met the following conditions, spontaneous respiration, intact airway reflexes, manageable airway secretion, and hemodynamic stabilization (9). The intensivist was responsible for the final decision to extubate and transfer the patients to the surgical ward. Postoperative mechanical ventilation duration, postoperative length of stay (LOS) in the ICU, and hospital were analyzed. Postoperative complications, readmission in 90 days, and in-hospital mortality were collected.

### 2.6. Follow-up

All patients were followed up in an outpatient clinic at regular intervals. They underwent physical exams, blood tests, and imaging tests. TTE was performed routinely before discharge and subsequently every three to six months.

### 2.7. Statistics

Results were expressed as the number (percentages) and mean (standard deviation) or median (range) when the distribution of variables was not normal. The Mann–Whitney test was used for continuous variables, and the *χ*^2^ test was used for categorical variables. A *p*-value less than 0.05 was considered statistically significant. Statistical analyses were performed using SPSS version 26.0 (SPSS Inc, Chicago, Illinois, USA).

## 3. Results

Patients' characteristics, as well as the demographic distribution, are listed in Table 1. There were 31 (28.2%) males and 79 (71.8%) females included in this series (*n* = 110), and the average age at admission was 54.05 ± 12.03 years (range: 19–78). Among these patients, 88 (80%) were in New York Heart Association class I–II, and 50 (45.5%) myxomas showed no symptoms, which were found occasionally. Meanwhile, 32 (29.1%) had dyspnea on admission, 15 (13.6%) palpitation, 11 (10%) exertional dyspnea, 5 (4.5%) chest pain, 3 (2.7%) dizziness, and 2 (1.8%) syncope. In some cases, constitutional findings were also reported, and these included non-specific symptoms such as fatigue (7.2%), anemia (0.9%), and fever (0.9%). Thromboembolic events occurred in 8 (7.3%) patients, with 5 (4.5%) patients having symptoms of cerebral thromboembolic events, 2 (1.8%) patients occluding the femoral artery, and 1 (0.9%) patient exhibiting signs related to the obstructive coronary artery.

**Table 1:** Patient characteristic (*n *= 110).

Variables	Results
Gender	
Male/Female	31 (28.2%)/79 (71.8%)
Age, years	54.05 ± 12.03
15–20	1 (0.9%)
21–30	2 (1.8%)
31–40	11 (10%)
41–50	20 (18.2%)
51–60	44 (40%)
61–70	27 (24.5%)
71–80	5 (4.5%)
BMI	24.47 ± 3.23
Symptoms	
Yes/No	60 (54.5%)/50 (45.5%)
Obstructive Symptoms	
Dyspnea	32 (29.1%)
Palpitation	15 (13.6%)
Exertional dyspnea	11 (10%)
Chest pain	5 (4.5%)
Dizziness	3 (2.7%)
Syncope	2 (1.8%)
Constitutional	
Fatigue	8 (7.2%)
Fever	1 (0.9%)
Anemia	1 (0.9%)
Embolic events	
Cerebral	5 (4.5%)
Peripheral	2 (1.8%)
Coronary artery	1 (0.9%)
NYHA functional class	
I–II	88 (80%)
III–IV	22 (20%)
Co-morbidities	
Hypertension	22 (20%)
Diabetes	8 (7.3%)
Coronary artery disease	4 (3.6%)
Valve disease	23 (20.9%)

BMI, body mass index; NYHA, new york heart association.

As shown in Table 2, patient preoperative variables were collected. Following an initial laboratory assessment of each patient, the erythrocyte sedimentation rate (ESR) and N-terminal pro-brain natriuretic peptide (NT-proBNP) were increased. In addition, electrocardiogram detected sinus tachycardia in 2 (1.8%) patients, bradycardia in 2 (1.8%), T wave changes in 8 (7.2%), non-specific ST segment changes were observed in 5 (4.5%) patients, and atrial arrhythmias and or fibrillations were found in 37 (33.6%) patients, premature ventricular contraction in 10 (9.1%). Moreover, pulmonary congestion was presented in 7 (6.4%) patients on chest x-ray (CXR) films.

**Table 2:** Preoperative variables and examination.

Variables	*n *= 110
Biochemical variables in blood	
White blood cell, ×10^9^/L	6.11 ± 1.49
Red blood cell, ×10^12^/L	4.42 ± 0.51
Hemoglobin, g/L	129.76 ± 17.27
Platelet, ×10^9^/L	260.09 ± 69.47
Prothrombin time, s	12.94 ± 0.66
ESR, mm/h	20.77 ± 23.16
NT-proBNP, pg/ml	300.76 ± 754.63
Electrocardiogram	
Tachycardia	2 (1.8%)
Bradycardia	2 (1.8%)
T wave change	8 (7.2%)
Non-specific ST segment changes	5 (4.5%)
Atrial arrhythmias or fibrillation	37 (33.6%)
Premature ventricular contraction	10 (9.1%)
CXR	
Enlargement of the left atrium	3 (2.7%)
Pulmonary congestion	7 (6.4%)
Transthoracic echocardiography	
Left ventricular ejection fraction, %	64.25 ± 4.42
Origin site of myxomas	
Left atrium	104 (94.5%)
Left atrium and ventricle	1 (0.9%)
Right atrium	6 (5.5%)
Shape of myxoma	
Solid/Papillary	104 (94.5%)/6 (5.5%)
Dimensions	
Largest diameter, cm	4.03 ± 1.52
Diastolic mobility	
No/Yes	22 (20%)/88 (80%)
Obstruction	
No/Yes	62 (56.4%)/48 (43.6%)
Co-Valve insufficiency	
Mitral regurgitation	16 (14.5%)
Aortic regurgitation	4 (3.6%)
Tricuspid regurgitation	21 (19.1%)
Pulmonary hypertension	11 (10%)

CXR, chest x-ray; ESR, erythrocyte sedimentation rate; NT-proBNP, N-terminal pro-brain natriuretic peptide.

By TTE, the mean ejection fraction value was 64.25% ± 4.42%. Left atrial myxomas were detected in 104 (94.5%) patients, whereas right atrial myxomas were detected in 6 (5.5%) patients, and 1 (0.9%) patient presented both left atria and left ventricle myxomas. The average dimension of myxomas was 4.03 cm ± 1.52 cm in the largest diameter. Eighty-eight (80%) cases showed diastole mobility, and 48 (43.6%) myxomas prolapsed into the ventricle. In addition, mild-to-severe mitral (*n *= 16), aortic valve (*n *= 4), and tricuspid (*n *= 21) incompetence or stenosis were detected.

Intraoperative data are reported in Table 3. Hemodynamic instability occurred in 38 (34.5%) patients after anesthesia induction; total crystalloid and or colloidal fluid infusion volume was 623 ± 319.27 ml, and intravenous infusion of dopamine (1–5 mg/kg/min) and or milrinone (0.2–1 mg/kg/min) occurred in 84 (76.4%) patients after rewarming. The duration of CPB was 63.35 ± 23.67 min; the clamp time was 39.05 ± 20.66 min. The myxomas ranged from the size of 1.0 × 0.9 × 0.7 × cm × cm × cm to 8.5 × 6.0 × 4.0 × cm × cm × cm, and the median volume was 40.62 cm^3^ ± 42.33 cm^3^, the mean weight was 23.07 ± 22.40 g. Routine pathological microscopy confirmed the diagnosis of myxoma in every instance.

**Table 3:** Intraoperative data.

Variables	*n *= 110
Hemodynamic instability during anesthesia induction	38 (34.5%)
Total infusion volume, ml	623 ± 319.27
Inotropic drugs after rewarming	84 (76.4%)
Heparin resistance	4 (3.6%)
Cardiopulmonary bypass	
CPB time, min	63.35 ± 23.67
XC time, min	39.05 ± 20.66
Size of myxoma	
Mean volume, cm^3^	40.62 ± 42.33
Mean weight, g	23.07 ± 22.40

CPB, cardiopulmonary bypass; XC, cross clamping.

**Figure 2:**
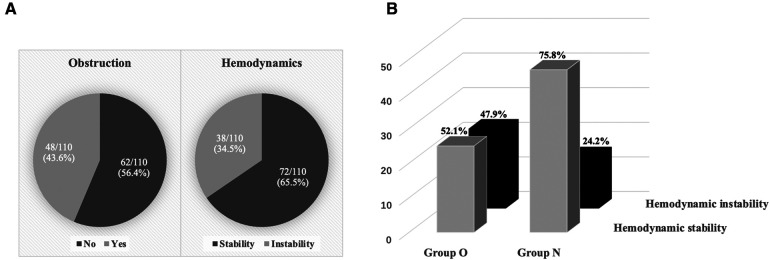
Hemodynamic status during anesthesia induction. (**A**) 48 (43.6%) myxomas obstructed the tricuspid or mitral valve, and 38 (34.5%) experienced hemodynamic instability during anesthesia induction. (**B**) Hemodynamic stability and hemodynamic instability between the two groups.

As shown in Figure 2, of the 110 patients, 48 (43.6%) myxomas prolapsed into the ventricle and obstructed tricuspid or mitral valve, which classified into group O, 38 (34.5%) experienced hemodynamic instability during anesthesia induction (Figure 2A). More patients in group O had hemodynamic instability (47.9% vs. 24.2%, *p* = 0.009) compared to group N (Figure 2B).

Obstructive myxoma characteristics are highlighted in Table 4. According to the preoperative TTE examination, 48 patients were finally divided into group O and 62 into group N. The NYHA class I–II was more prevalent in group N. However, in group O, palpitation and valve disease were found to be more prevalent, ESR and NT-proBNP significantly increased, and myxoma size was more significant. All seven pulmonary congestion cases occurred in group O. Moreover, higher diastolic mobility myxomas were presented in group O (100%) than in group N (64.5%), which contributed to tricuspid regurgitation (29.2% vs. 11.3%, *p* = 0.018) and hemodynamic instability (47.9% vs. 24.2%, *p* = 0.009).

**Table 4:** Comparison between two groups.

Variables	Group N (*n *= 62)	Group O (*n *= 48)	*p*
Symptoms			
Palpitation	4 (6.5%)	11 (22.9%)	0.027
NYHA functional class			
I–II	57 (91.9%)	31 (64.6%)	0.000
III–IV	5 (8.1%)	17 (35.4%)	0.000
Co-morbidities			
Valve disease	7 (11.3%)	16 (33.3%)	0.005
Biochemical variables in blood			
Hemoglobin, g/L	133.69 ± 15.89	124.69 ± 17.80	0.01
Platelet, ×10^9^/L	248.38 ± 65.12	275.21 ± 72.63	0.041
ESR, mm/h	14.43 ± 15.76	28.76 ± 28.21	0.002
NT-proBNP, pg/ml	83.21 ± 94.50	560.87 ± 1,061.95	0.000
CXR			
Pulmonary congestion	0	7 (14.9%)	0.006
Transthoracic echocardiography			
Largest diameter, cm	3.13 ± 1.02	5.20 ± 1.25	0.000
Diastolic mobility	40 (64.5%)	48 (100%)	0.000
Tricuspid regurgitation	7 (11.3%)	14 (29.2%)	0.018
Hemodynamic instability during anesthesia induction	15 (24.2%)	23 (47.9%)	0.009

CXR, chest x-ray; ESR, erythrocyte sedimentation rate; NT-proBNP, n-terminal of the prohormone brain natriuretic peptide; NYHA, New York heart association.

**Figure 3:**
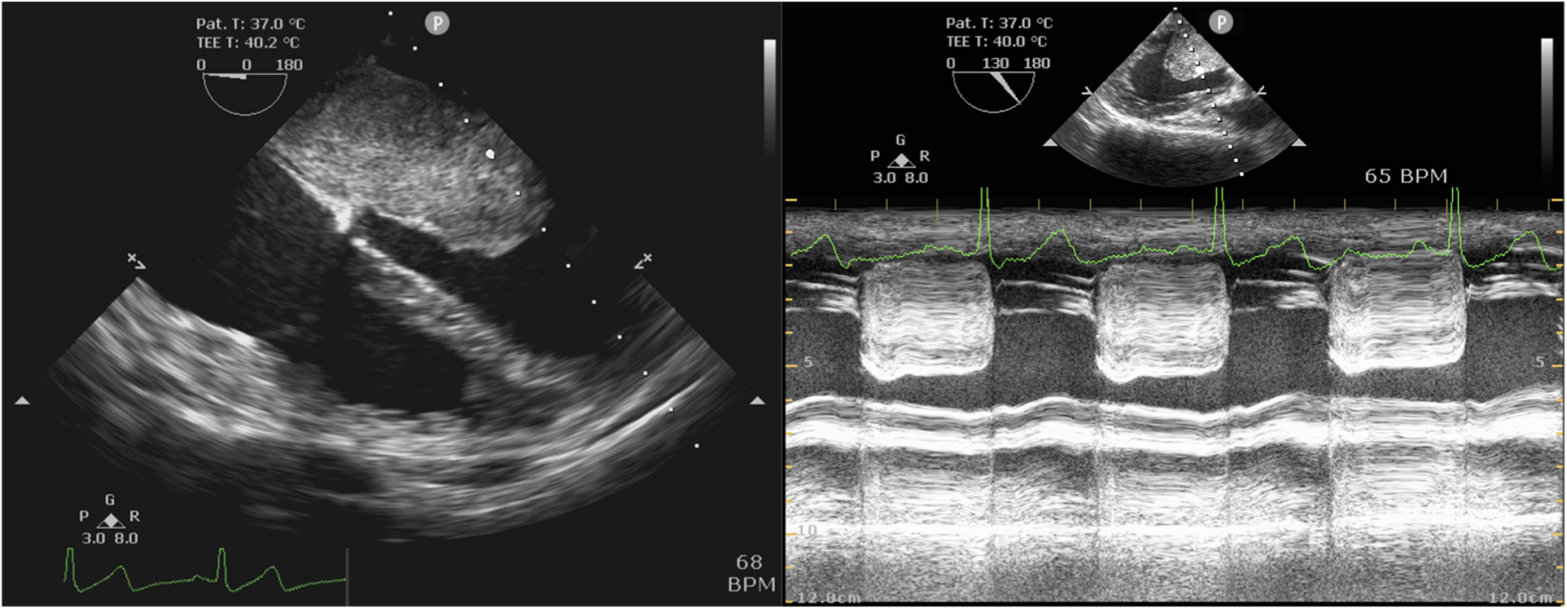
Intraoperative TEE examination. Left: Mid-esophageal four-chamber view showed the left atrial myxoma. Right: Mid-esophageal long-axis view by M mode demonstrated mitral obstruction.

TEE revealed a mass attached to the interatrial septum in the left atrium, which obstructed the mitral orifice during diastole (Figure 3). As shown in Table 5, the mean postoperative mechanical ventilation duration was 12.52 ± 4.69 h, the mean LOS in the ICU was 41.97 ± 27.21 h, and the hospital 10.64 ± 3.01 days. In-hospital mortality did not occur in any of the cases analyzed. However, several postsurgical complications were reported. These included: atrial fibrillation (*n *= 1), incision infection (*n *= 1), pericardial effusion (*n *= 1), and postoperative bleeding which had reoperation experience (*n *= 1). Moreover, three patients underwent hospital readmission within 90 days after discharge. The reasons for hospital readmission varied from atrial flutter and persistent fever to pericardial effusion.

**Table 5:** Postoperative data.

Variables	*n *= 110
Mechanical ventilation duration, hours	12.52 ± 4.69
LOS in the ICU, hours	41.97 ± 27.21
LOS in the hospital, days	10.64 ± 3.01
Postoperative complications	
Atrial fibrillation	1 (0.9%)
Incision infection	1 (0.9%)
Pericardial effusion	1 (0.9%)
postoperative bleeding reoperation	1 (0.9%)
90 days readmission	3 (2.7%)
In-hospital mortality	0

LOS, length of stay; ICU, intensive care unit.

## 4. Discussion

This study comprehensively summarized the anesthetic management for myxoma excision. Cardiac myxomas are the most common primary cardiac tumors (3, 10). It is estimated that more than 75% of myxomas originate in the left atrium; 20% arise from the right atrium, while 5% stem from both atria and the ventricle (3). Atrial myxomas occur predominantly in females, and most cases occur between the fourth and sixth decade of life (3). Compared with the previous study of cardiac myxoma diagnosed in China, the present study was similar in terms of age, sex, and tumor location (11), and it was similar to the populations from Turkey and Greece (12, 13).

As a great imitator, cardiac myxoma, even a benign tumor, can cause serious consequences (4), highlighting that anesthetic management is vital for patients undergoing primary cardiac myxoma resection.

In preoperative evaluation, clinical features in the collected series of atrial myxoma reveal that dyspnea is the most common symptom (3, 11–13). However, unlike other studies of cardiac myxoma cases, this series identified that 50 (45.5%) myxomas showed no symptoms at all, which attributed to more widespread multimodality imaging and the health insurance schemes extending coverage in China (14). Therefore, the earlier myxomas were detected, the more patients were in NYHA classes I–II. Due to the high systolic pressure and location, left atrial myxomas were highly associated with an increased risk of systemic embolization, particularly in the central nervous system (15). Hence, the neurologic function must be carefully assessed, and fast-track cardiac anesthesia is safe and effective (16). Eight thromboembolic cases were under intervention before myxoma resection, and the outcome was excellent.

As in most series, high ESR was the most common laboratory finding, with a mean of 20.77 ± 23.16 s. When patients do not present with sufficient signs or symptoms for a clinician to make a definitive diagnosis, echocardiography is usually the diagnostic modality of choice (17). For example, tumor location, size, attachment, mobility, and the extent to which the tumor can obstruct circulation, as well as considerations regarding the decision to perform surgical excision, can be assessed using this technique (18). Therefore, anesthesia providers must review all echocardiography details to formulate a good anesthetic plan and pay attention to the myxoma prolapsed into the ventricular and obstructed tricuspid or mitral valve.

Depending on size and mobility, atrial myxomas commonly give rise to signs of obstructed filling of the left or right ventricle with subsequent dyspnea, recurrent pulmonary edema, congestive heart failure, and even death (6). These signs mimic the clinical picture of mitral- or tricuspid-valve stenosis (1). As they mimic mitral stenosis, it is crucial to avoid tachycardia because a rapid heart rate could decrease diastolic filling time and increase the pressure gradient across the mitral valve, further increasing pulmonary artery pressure (19, 20). As in most series, dyspnea and palpitation were the most common presentations in our case, and palpitation especially prevailed in group O. Hence, beta-blockers atenolol 2–5 mg might be used to alleviate tachycardia before anesthesia induction.

Anesthetic considerations in patients with right atrial myxoma include hypoxemia, low cardiac output, possible right-to-left shunt, and potential paradoxical embolization (21). Pulmonary artery catheterization (PAC) may be contraindicated (22). There was no PAC insertion in our series. Meanwhile, the central venous catheter (CVC) placement needed to be cautious and weighted against potential embolization by myxoma during catheter insertion. Intraoperative CVC placement can be guided by TEE to prevent further obstruction of the tricuspid valve or cardiovascular collapse from embolization (22, 23).

Typically, tricuspid or mitral valve with obstruction is a premier ingredient for perioperative and anesthetic management, so we observed the differences between group O and group N. Partial obstruction of the mitral valve resulted in pulmonary vein congestion and pulmonary edema, elevating pulmonary artery pressure, and reducing cardiac output (24). In our study, all seven pulmonary congestion cases were in group O. This series identified that myxomas in group O were giant with a mean largest diameter of 5.2 cm ± 1.25 cm than 3.13 cm ± 1.02 cm in group N, which provides anesthetic implications to avoid hemodynamic instability and lethal events have been shown for the intraoperative management of patients undergoing excision of a giant obstructive cardiac myxoma (25).

Induction of general anesthesia in patients with myxomas should be performed cautiously with the following goals: constant monitoring of systemic blood pressure, adequate IV fluids, and judicious use of vasoactive medications to prevent a fall in systemic vascular resistance (7). There were three main treatments to avoid hemodynamic instability. Firstly, 100–200 ml of crystalloid was administered rapidly to maintain preload. Secondly, phenylephrine 50 mg or methoxamine 1 mg was injected intravenously. Thirdly, Trendelenburg position helped relieve the valvular obstruction and contributed to the redistribution of pooled venous blood from the lower limbs back to the heart by increasing preload and cardiac output (19, 20, 25, 26).

In patients undergoing cardiopulmonary bypass, heparin resistance is often defined as the need for a dose of more than 500 U per kilogram of body weight to achieve an ACT of 400–480 s (27). Antithrombin III deficiency is a commonly implicated cause of heparin resistance (28). In this series, 4 cases were presented with heparin resistance. When heparin resistance coincides with profound cardiovascular instability, it requires a pragmatic response to expedite the establishment of cardiopulmonary bypass while minimizing potential harm (29). Therefore, it highlights the importance of anesthetic management for the potential heparin resistance scenario.

TTE is a reliable method in diagnosing atrial myxomas, but not in all cases, such as emphysema, obesity, or chest deformity patients. At the same time, TEE has been found to always be reliable and valuable in assessing atrial myxoma (18, 30). Therefore, given the importance and utility of TEE perioperatively in patients undergoing myxoma resection, pre-CPB TEE was routinely performed, which helped confirm the diagnosis and monitoring and guide surgical resection. Post-CPB TEE would provide an assessment of valve and cardiac function following resection. Like many other studies (1, 7, 31, 32), we consider intraoperative TEE to be an indispensable instrument in the anesthesia management of cardiac myxoma resection.

Surgical removal of cardiac myxoma should be performed promptly when the diagnosis is made to prevent complications such as embolism and sudden death (1, 6). The primary intracardiac myxoma resection was performed under general anesthesia in all patients timely. Most patients made an uneventful postoperative recovery, the postoperative complications were not anesthesia-related, and the mean LOS in the hospital was 10.64 ± 3.01 days. In our study, three (2.7%) patients underwent recurrence. Meanwhile, in the Chinese population, the recurrence rate for myxomas is 3% (33). In this series, the anesthetic management of these patients was safe and effective.

Although this study is valuable because it summarizes the experiences of anesthetic management in cardiac myxoma resection in the Chinese population, there were some limitations worthy of note. Firstly, it was a single-center retrospective study which might have patient selection bias. Secondly, although we reviewed 110 cases in the last three years, it might still be insufficient due to heterogeneity resulting from different anesthesiologists, surgeons, and patients. Finally, concomitant surgery, such as tricuspid or mitral valvoplasty, mitral or aortic valve replacement, and coronary artery bypass grafting, did not occur in this study, which might challenge anesthetic management.

In conclusion, we summarize our anesthetic management experiences in primary cardiac myxoma resection. Based on these considerations and experiences, we recommend reviewing the echocardiography details attentively in the preoperative evaluation and paying attention to the myxoma prolapsed into the ventricle, especially the obstructive myxoma. The prevention of hemodynamic instability is imperative during anesthetic management. Moreover, specific concerns should be raised on obstructive myxomas because they make a difference.

## Data Availability

The raw data supporting the conclusions of this article will be made available by the authors, without undue reservation.
